# Co-expression of PD1+ and HLA-DR+ in CD8+ T cells is increased in tonsils of children with EBV primary and persistent infection

**DOI:** 10.3389/fimmu.2025.1653165

**Published:** 2025-09-02

**Authors:** María Eugenia Amarillo, Karen Lindl, Veronica Lapido, Ignacio E. Rojas Campión, M. Soledad Collado, Johanna Speratti, Andrea Valerio, Plácida Baz, Elena De Matteo, L. Ariel Billordo, Paola Chabay

**Affiliations:** ^1^ Multidisciplinary Institute for Investigation in Pediatric Pathologies (IMIPP), National Council for Scientific and Technological Research (CONICET)-GCBA, Laboratory of Molecular Biology, Pathology Division, Ricardo Gutiérrez Children’s Hospital, Ciudad Autónoma de Buenos Aires, Argentina; ^2^ Institute of Immunology, Genetics and Metabolism (INIGEM), Clinical Hospital ‘José de San Martín’, University of Buenos Aires (UBA), National Council for Scientific and Technological Research (CONICET), Ciudad Autónoma de Buenos Aires, Argentina; ^3^ Otorhinolaryngology Division, Ricardo Gutierrez Children Hospital, Ciudad Autónoma de Buenos Aires, Argentina; ^4^ Pathology Division, Ricardo Gutierrez Children Hospital, Ciudad Autónoma de Buenos Aires, Argentina

**Keywords:** EBV, tonsil, children, CD4+ T cell, CD8+ T cells

## Abstract

**Introduction:**

Epstein–Barr virus (EBV) infects B lymphocytes and establishes lifelong persistence in the B cells. While systemic T-cell responses have been well characterized, the local immune response at the site of viral entry in children from undeveloped countries remains poorly understood.

**Methods:**

Tonsillar CD4 and CD8 T cells in 32 pediatric patients undergoing tonsillectomy were classified as primary infected (PI), EBV carriers (EC), and non-infected children by serology. T-cell subsets were assessed by flow cytometry, whereas LMP1 and EBNA2 viral proteins were evaluated by immunohistochemistry.

**Results:**

A higher percentage of activated HLA-DR+ CD8 T cells in PI patients was demonstrated. Notably, PD-1 expression was increased in both PI and EC, in particular in activated HLA-DR+ CD8 T cells. Positive correlations of EBNA2 with follicular helper T cells and Th1 cells, as well as a negative correlation between EBNA2 and activated CD8 T cells, were observed.

**Discussion:**

These findings suggest that, during asymptomatic primary infection by EBV, activated CD8 T cells are observed, but they may be cells that may exhibit features of exhaustion, which probably explains the absence of symptoms. PD-1 expression in CD8 T cells remains in EC. Additionally, Tfh, Th1, and CD8 T cells may influence the expression of EBNA2 and LMP1 latent viral antigens in tonsils.

## 1 Introduction

Epstein–Barr virus (EBV) is a ubiquitous γ-herpesvirus that infects more than 90% of adults globally (1). EBV infection is mostly asymptomatic in children; however, in adults, it can present as infectious mononucleosis (IM). The virus is transmitted through saliva from carriers and, upon entering the oropharyngeal region, reaches the primary site of infection and reactivation, the tonsils, where its main target, the B lymphocyte, resides (2). As a member of the herpesvirus family, EBV displays a dual-phase life cycle, which consists of latent and lytic stages, both fundamental to its pathogenicity (3). Following primary infection, EBV predominantly persists in long-lived memory B cells. In order to reach this reservoir, EBV initially infects naive B cells, promoting their proliferation and the expression of the Latency III program, characterized by the expression of EBNA1, 2, 3A, 3B, 3C, LP, LMP1, 2A, 2B, and the EBERs and BART RNAs. These infected B cells then migrate to the germinal center (GC), where they downregulate the expression of most EBNA antigens, except for EBNA1, to facilitate the transition to the Latency II program. Upon exiting the GC, EBV-infected cells differentiate into memory B cells, adopting the Latency 0 (L0) program, characterized by complete viral gene silencing, except for EBERs and BARTs, to evade detection by EBV-specific immune cells. Over time, proliferating EBV-infected memory B cells can switch to the Latency I (LI) program, in which EBNA1 facilitates the segregation of the viral episome into daughter cells (4).

Since its discovery in 1964, EBV has been associated with a wide range of cancers worldwide. In fact, it is estimated that EBV was linked to approximately 1.3%-1.9% of the global cancer burden (5). In 1997, the International Agency for Research on Cancer (IARC) classified EBV as a Group 1 carcinogen due to its causal link to endemic Burkitt lymphoma (eBL), Hodgkin lymphoma (HL), and nasopharyngeal carcinoma (NPC) (6). More recently, EBV has also been implicated in cancers such as extranodal NK/T-cell lymphoma, nasal type (ENKTL-NT), certain subtypes of diffuse large B-cell lymphoma (DLBCL), and gastric carcinoma (GC) (5). Moreover, recently EBV has been linked to autoimmune diseases, primarily multiple sclerosis (MS) (7). Most types of cancer take many years to develop, and the fact that EBV infection persists in the long term gives the possibility of a sustained contribution to cancer development, but only in a small subset of people (1). The rare occurrence of virus-induced diseases in otherwise healthy infected individuals may be due to a strong immune response against EBV, primarily driven by EBV-specific CD8+ and CD4+ T cells.

Primary EBV infection induces NK cell activation, in particular in children, large expansions of virus-specific CD8+ T cells, and smaller expansions of virus-specific CD4+ T cells in the blood (8). High titers of EBV-specific CD8+ CTLs have been found in samples from IM patients, both in lymphoid cells (specifically Waldeyer’s ring) and in peripheral circulation, whereas persistent EBV infection has been shown to be controlled also, at least in part, by CD8+ EBV-specific CTLs. The CD4+ T-cell response to EBV is less robust than the CD8+ T-cell response but exhibits greater variability. Cytotoxic CD8+ T-cell responses predominantly target viral lytic cycle gene products, whereas CD4+ T-cell response seems to be spread more evenly across the whole range of available lytic cycle as well as latent antigens (9). In mice with a humanized immune system, CD8+ tissue-resident memory T cells expressed canonical markers of activation, but they failed to control EBV local viral loads in primary infection. Alternatively, systemic CD8+ T-cell expansion seems to control viral loads in the context of IM-like infection (10). Conversely, local immune response seems to play a key role during persistent infection. In fact, after resolution of IM, EBV-specific CD8+ T cells begin to return to a resting state, and these T cells are recruited to the tonsil to control EBV-induced growth transformation of B cells (11). However, the scenario of the local CD4+ T-cell immune response, particularly within germinal centers, the histological site where most lymphomas originate, has not yet been fully elucidated.

In Argentina, EBV infection is usually asymptomatic, with around 90% of individuals achieving seroconversion by the age of 3. Furthermore, a statistical correlation has been identified between EBV and B-cell lymphoma in patients younger than 10 years, suggesting that early EBV seroconversion may be associated with an increased risk of developing B-cell lymphoma (12). The origin of EBV-associated malignancies may be related to a disruption in the balance of the immune response. In fact, in EBV-driven malignancies, the tumor microenvironment is modulated for viral benefit. To investigate whether the characteristics of the local immune response in children from Argentina might ultimately be linked to an increased susceptibility to developing EBV-associated lymphomas, the aim of this study is to characterize the total CD4+ and CD8+ T-cell responses in children with primary and persistent EBV infection.

## 2 Materials and methods

### 2.1 Patients and samples

A total of 32 pediatric patients aged between 2 and 13 years old (median and mean 7) undergoing tonsillectomy due to non-reactive hyperplasia at the Otorhinolaryngology Division, Ricardo Gutierrez Children’s Hospital (Buenos Aires, Argentina) were enrolled in this study. All samples were collected after written consent (for patients older than 12 years of age and legal guardians of children younger than 12 years of age) and assent (7- to 12-year-old patients and legal guardians of children older than 12 years of age). This study was approved by the Ethical Committee of the Ricardo Gutiérrez Children’s Hospital, in accordance with the Helsinki Declaration of 1975. Fresh and formalin-fixed paraffin-embedded (FFPE) tonsil samples were processed and obtained at the Pathology Division of Ricardo Gutierrez Children’s Hospital (Buenos Aires, Argentina).

### 2.2 Immunoassays for Epstein–Barr virus serology

A blood sample was taken from the patient at the time of tonsillectomy to assess the serologic profile for EBV. EBV serology was performed in the serum using an ELFA (enzyme-linked fluorescence assay) with the Panel VIDAS^®^ EBV (bioMérieux) to assess the presence of antibodies in serum: VCA-IgG/EA-IgG, VCA-IgM, and EBNA1-IgG. Patients with primary infection (PI) were defined by the presence of VCA-IgM and VCA-IgG antibodies; EBV carrier patients (EC), by VCA-IgG and EBNA1-IgG presence and non-infected patients (NI) by the absence of EBV antibodies.

### 2.3 Flow cytometry

A fresh piece of tonsil was mechanically disaggregated with phosphate-buffered saline (PBS)–bovine serum albumin (BSA) 1% (stain buffer) and filtered, and cells obtained from the suspension were counted. The cells were resuspended in a stain buffer in a final concentration of 10^6^ cells per 100 µl. The suspension was stained with different panels of fluorochrome-conjugated monoclonal antibodies shown in the **Supplementary Table** (**Supplementary Table 1**). These antibodies were previously titrated to define their optimal concentration. Briefly, the staining with monoclonal antibodies against surface markers was incubated for 30 min at 4 °C in the dark, fixed with fixation buffer (BioLegend), and the cells were acquired the following day. Concerning intracellular staining of FOXP3, it is first incubated for 30 min with the surface markers at 4 °C in the dark and then fixed and permeabilized with the FOXP3 Fix/Perm Buffer Set (BioLegend) according to the manufacturer’s instructions and was incubated for 30 min at 4 °C in the dark with anti-FOXP3-Alexa Fluor 647 antibody. Finally, samples were acquired on a FACSAria II (BD Biosciences, CA, USA) with BD FACSDiva software. Automated compensation was calculated using COMPBeads (BD Biosciences) on BD FACSDiva software. Flow cytometry data were analyzed in FlowJo v10, and percentage of different T-cell subpopulations evaluated in this study were obtained.

Fluorescence Minus One controls (FMO) were added to the assay in order to discriminate positive from negative subpopulations, especially for those markers with a continuous expression pattern such as CXCR3, CCR6, HLA-DR, and CD27 (**Supplementary Figure 1**). FMO were prepared staining the samples with the full Panel except that one marker whose signal was needed to resolve better.

### 2.4 EBERs *in situ* hybridization and immunohistochemistry for viral antigens

EBER *in situ* hybridization (ISH) was performed using fluorescein isothiocyanate (FITC)-conjugated EBER oligonucleotides as probes (Dako, Carpinteria, CA, USA). A monoclonal antibody anti-FITC labeled with alkaline phosphatase was used for the detection of hybridized sites (Dako), with NBT-BCIP as a substrate for the enzyme, according to the manufacturer’s instructions as described previously (13).

Immunohistochemistry (IHC) was performed in FFPE tonsil biopsy samples cuts (3-4 µm). Primary antibodies for LMP1 (CS1–4 pool of clones, Dako) were used to evaluate the expression of this latency antigen as described previously (14). The primary antibody for EBNA2 (PE2 clone, Abcam) was used to evaluate the expression of EBNA2. Briefly, the antigenic retrieval with sodium citrate buffer (pH 6) in an autoclave oven for 10 min was performed.

A 1/300 dilution of the primary antibody was used and incubated overnight at 4 °C. IHC detection was carried out using a universal streptavidin–biotin complex-peroxidase detection system (VECTASTAIN^®^ Elite^®^ ABC-HRP Kit) according to the manufacturer’s instructions.

Patients were classified and grouped into latency profiles according to their expression of the following viral antigens: Latency 0/I (EBERS+), Latency II (EBERS+ and LMP1+), Latency IIb (EBERS+ and EBNA2+), and Latency III (EBERS+, LMP1+ and EBNA2+).

### 2.5 Quantification for viral antigens

The tonsil was completely explored using a 100× objective, and four histological regions were evaluated: the subepithelial zone (SE), germinal center (GC), mantle (M), and interfollicular region (IF). The study also considered the total counts of each latency protein, LMP1 and EBNA2, referred to as LMP1 total (T) and EBNA2 T, respectively.

The pictures were taken with a 100× objective using a ZEN 3.6 (blue edition) imaging platform, with an A1 Axio Scope (Carl Zeiss) microscope. The number of positive cells per cm^2^ was quantified for each histological region. The percentage of expression of each viral latency antigen in different histological regions was calculated, including only patients who showed expression of the evaluated viral antigen.

### 2.6 Statistical analysis

Statistical tests were performed using Prism 9.4.1 (GraphPad Software). Normality test was applied using the Shapiro–Wilk test. The *t*-test or Mann–Whitney (MW) test according to the normality test results was performed to compare means between two groups. Mean comparison of more than two groups was assessed by one-way ANOVA or Kruskal–Wallis (KW) test according to the normality test results, followed by Tukey’s multiple comparisons and Dunn’s multiple comparisons tests, respectively. Correlations were assessed using Spearman or Pearson tests, when appropriate. Outliers were defined using the Robust test to compare data median absolute deviation (Mad) in Excel. All tests were two-tailed, and p < 0.05 was considered statistically significant.

The results were graphically represented by violin plots, where the distribution of the data can be observed, and the median is defined as a solid line, whereas the quartiles are represented as a dotted line. The color of each violin represents a specific group of patients studied. Gray was assigned to the PI group, turquoise to the EC, and blue to the combined EBV-infected group, including PI and EC. Finally, pink was assigned to the NI group (EBV−).

## 3 Results

### 3.1 Latency profiles and EBV-infection status

**Figure 1:**
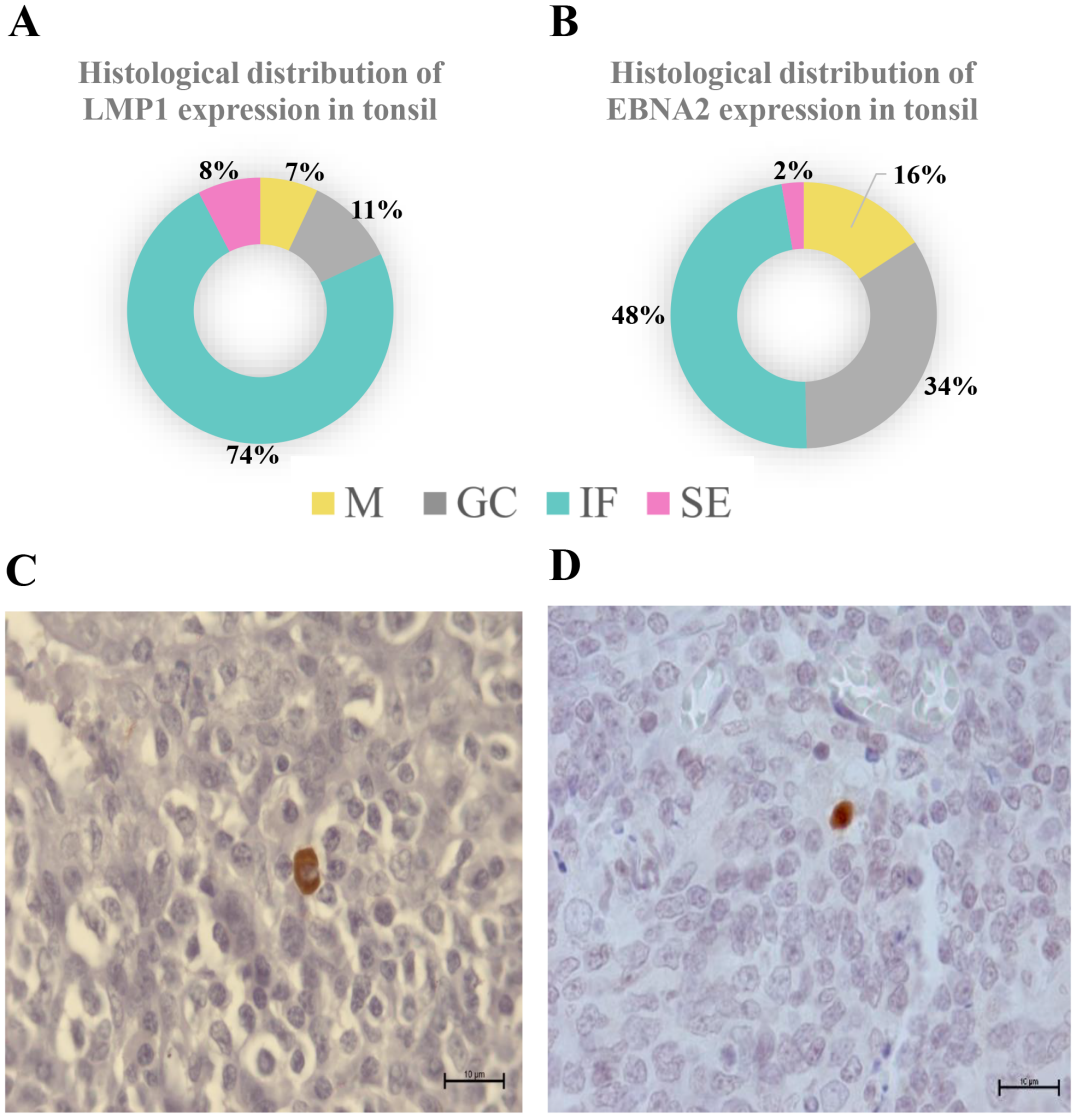
Histological distribution of viral latency proteins expression in tonsil. Histological distribution of LMP1 **(A)** and EBNA2 **(B)**. A representative picture of LMP1 **(C)** and EBNA2 **(D)** expression is shown in the lower panel at 1,000×. M, mantle; GC, germinal center; IF, interfollicular; SE, subepithelial.

In our cohort of 32 pediatric patients, 27 patients were EBV-infected (21 EC and 6 PI) and 5 patients were NI. Concerning latency profiles, 7 patients displayed latency 0/I, 4 latency II, 2 latency IIb, and 14 latency III. Latency profiles according to EBV infection status are shown in **Supplementary Table 2**. As previously observed, latency III was demonstrated in PI as well as in EC (14). When the histological distribution of viral proteins was evaluated, most LMP1 proteins were expressed at the IF region (**Figure 1A**). In contrast, EBNA2 protein was expressed in similar percentages between the GC and IF regions (**Figure 1B**).

### 3.2 The T-cell distribution in different EBV infection statuses

**Figure 2:**
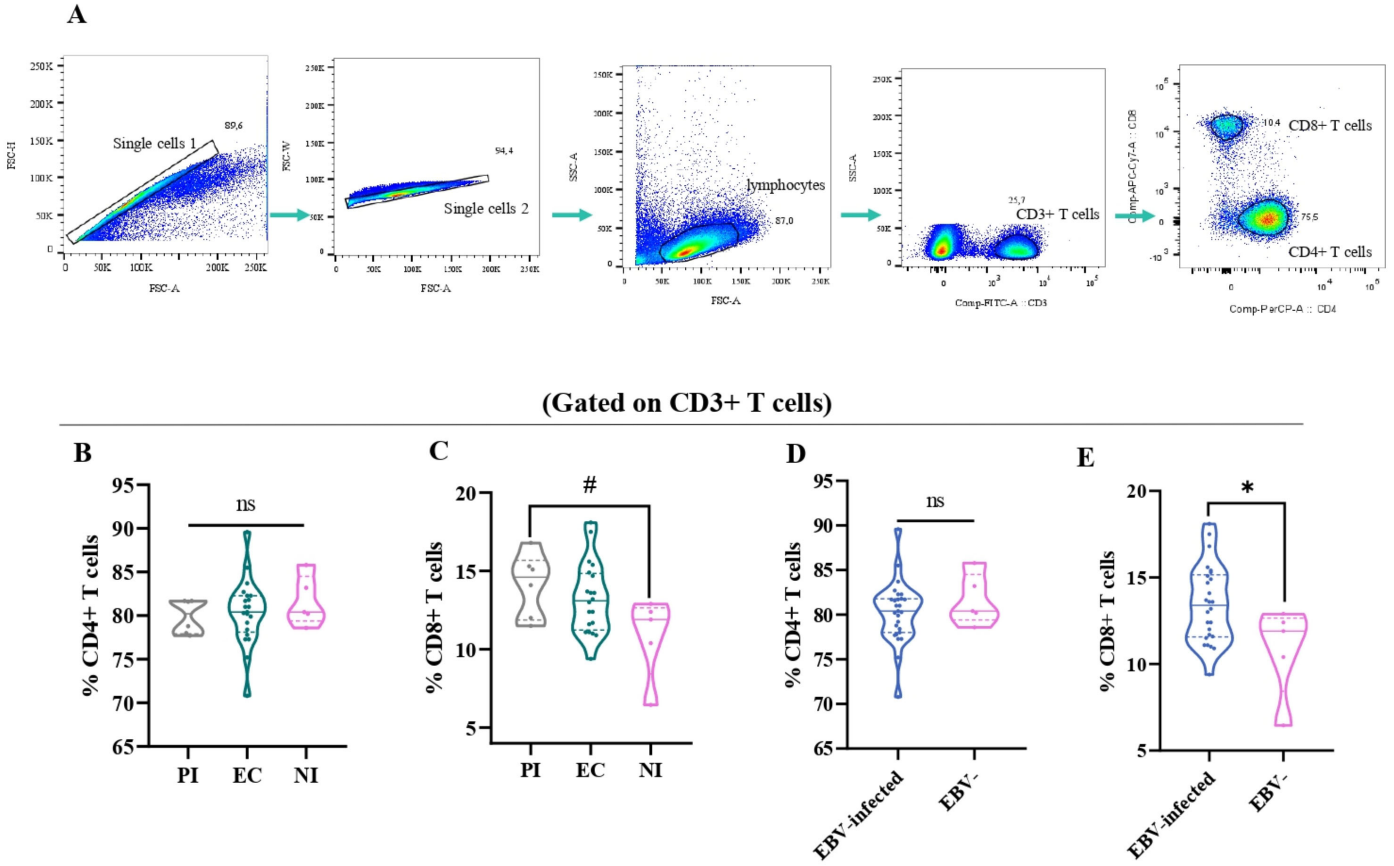
The T-cell distribution in different EBV infection status. Representative gating strategy for analyzing CD4+ and CD8+ T cells on CD3+ T cells **(A)**. The percentage of CD4+ **(B)** and CD8+ T cells **(C)** in primary infected (PI) patients, EBV carriers (EC), and non-infected (NI) are represented by violin plots. The percentage of CD4+ **(D)** and CD8+ T cells **(E)** according to EBV-infection status: EBV-infected (EI) (PI+EC) patients are represented by violin plots. # Trend *p<0.05, ns: no significant.

A comparative analysis was performed among EC, PI, and NI to assess differences in the percentages of CD4+ and CD8+ T cells based on EBV infection status. The gating strategy used to determine these cell percentages is detailed in **Figure 2A**.

Consistent with our previous findings on immunostaining in tonsils, there were no significant differences in the percentage of CD4+ T cells among the different infection statuses (p>0.05, ANOVA) (**Figure 2B**) (15). However, when the percentage of CD8+ T cells among the different EBV infection statuses was evaluated, a trend toward a higher percentage of CD8+ T cells in PI compared with NI was demonstrated (p=0.0587, ANOVA followed by Tukey test) (**Figure 2C**). When considering EBV-infected+ children compared with NI ones, only the percentage of CD8+ T cells was significantly higher in EBV-infected patients than in NI patients (p=0.0244, *t*-test) (**Figures 2D, E**).

### 3.3 The percentage of activated CD8+ T cells was higher in PI

**Figure 3:**
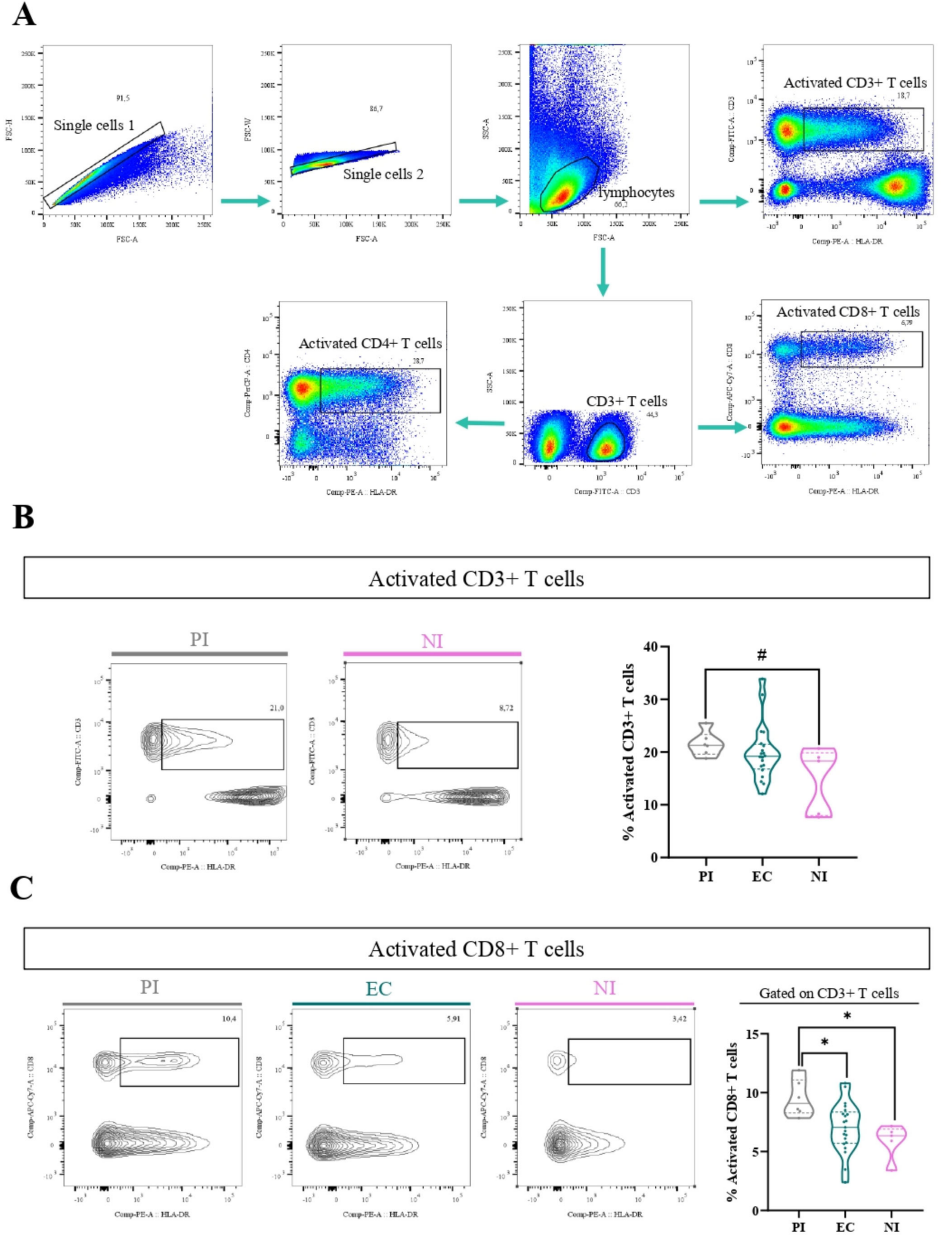
Activated T cells. Representative gating strategy for analyzing activated CD3+ (CD3+ HLA-DR+), CD4+ (CD3+ CD4+ HLA-DR+), and CD8+ (CD3+ CD8+ HLA-DR+) T cells **(A)**. Representative contour plots of activated CD3 T cells for PI patients and NI and violin plot on the right of activated CD3 T cells for PI patients, EC, and NI **(B)**. Representative contour plots of activated CD8+ T cells for PI patients, EC, and NI and violin plot on the right of activated CD8 T cells for PI patients, EC, and NI **(C)**. # Trend *p<0.05, **p<0.01, ***p<0.001.

In order to determine the activation of different T-cell populations, the expression of the HLA-DR marker was assessed by flow cytometry. The gating strategy to analyze the percentage of these cells is shown in **Figure 3A**. When activated CD3+ T cells (CD3+ HLA-DR+) were explored, there was a trend to a higher frequency of activated CD3+ T cells in PI compared with NI (P=0.0723, Kruskal–Wallis, KW, followed by Dunn’s test) (**Figure 3B**). When deeper analysis was assessed to explore the specific T cells contributing to this increase in PI, activated CD8+ T cells (CD8+ HLA-DR+) were higher in the PI compared with NI (p=0.0110, ANOVA, followed by Tukey test) and EC (p=0.0233, ANOVA, followed by Tukey test) (**Figure 3C**), whereas no differences were observed in activated CD4+ T cells (CD4+ HLA-DR+) in the different EBV infection statuses (p>0.05, KW test). In contrast, when considering EBV-infected in relation to NI patients, no differences were observed in activated CD3+, CD8+, and CD4+ T cells (p>0.05, t-test).

### 3.4 The percentage of PD-1+ CD8+ T cells was higher in EBV-infected patients

**Figure 4:**
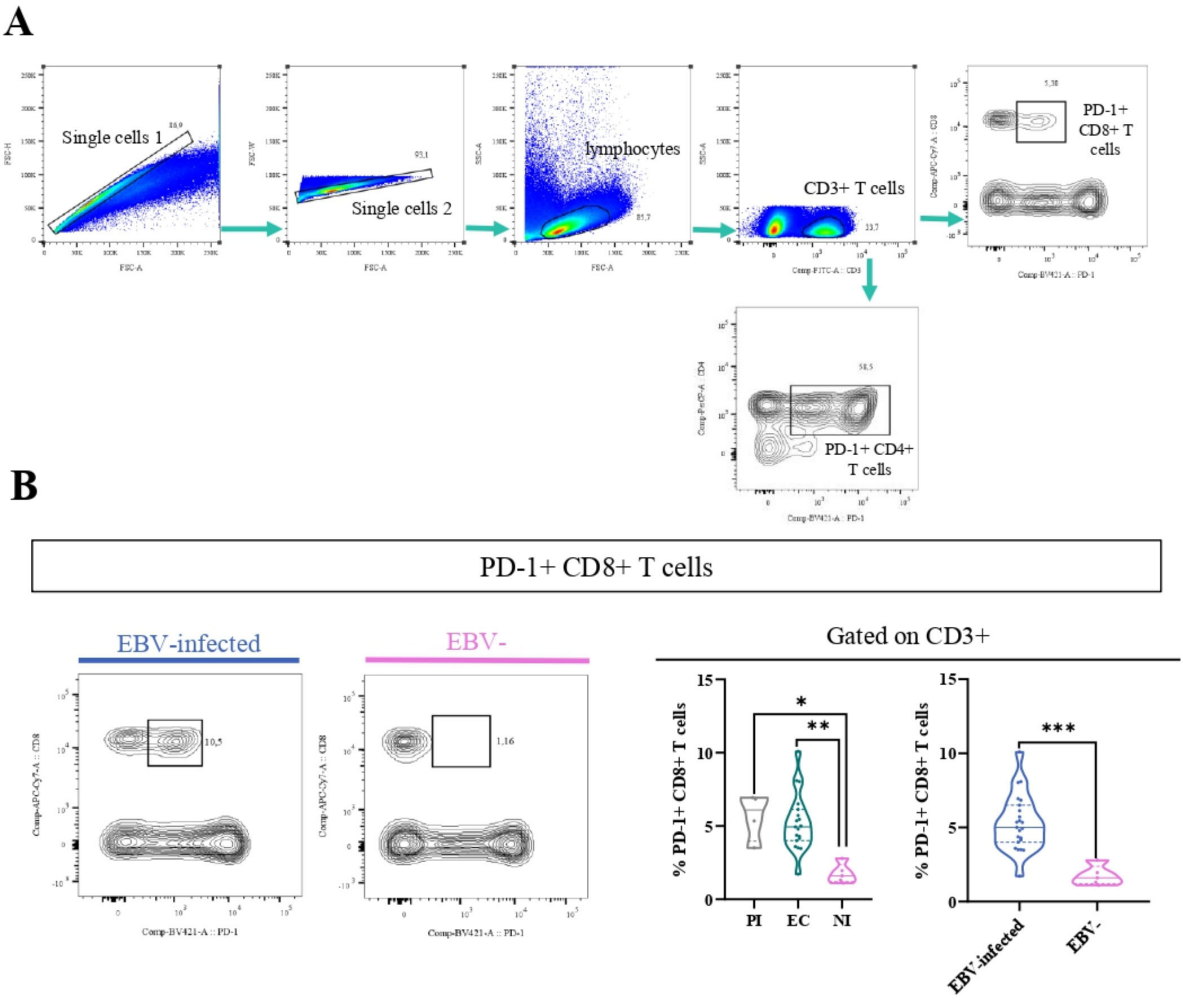
PD-1+T cells. Representative gating strategy for analyzing PD-1+CD4+ and CD8+ T cells **(A)**. Representative contour plots on the left of PD-1+CD8 T cells for EBV + patients and EBV−, and violin plots on the right of PD-1+CD8+ T cells for PI patients, EC and NI, and EBV-infected (EI) and non-infected (NI) patients **(B)** *p<0.05, **p<0.01, ***p<0.001.

It is well-known that in both chronic viral infections and cancer, there is an increase of PD-1+CD8+ T cells (16). Therefore, to explore this issue in the context of asymptomatic EBV infection in children, the percentage of PD-1+ cells by the expression of the PD-1 marker in T cells at the tonsils, the entry site of the EBV infection, was performed. The gating strategy to analyze the percentage of these cells is shown in **Figure 4A**. Remarkably, PD-1+CD8+ T cells (CD3+CD8+PD-1+) were higher in EBV-infected patients than in NI patients (p=0.0003, *t*-test). Furthermore, when EBV status infection was more deeply evaluated, the frequency of PD-1+CD8 T cells was statically lower in NI than in EC and PI (p=0.0046 and p=0.0151, KW followed by Dunn’s test, respectively) (**Figure 4B**). Conversely, no differences were demonstrated in PD-1+CD4+T cells (CD3+CD4+PD-1+) in the different EBV infection status (p>0.05, KW).

**Figure 5:**
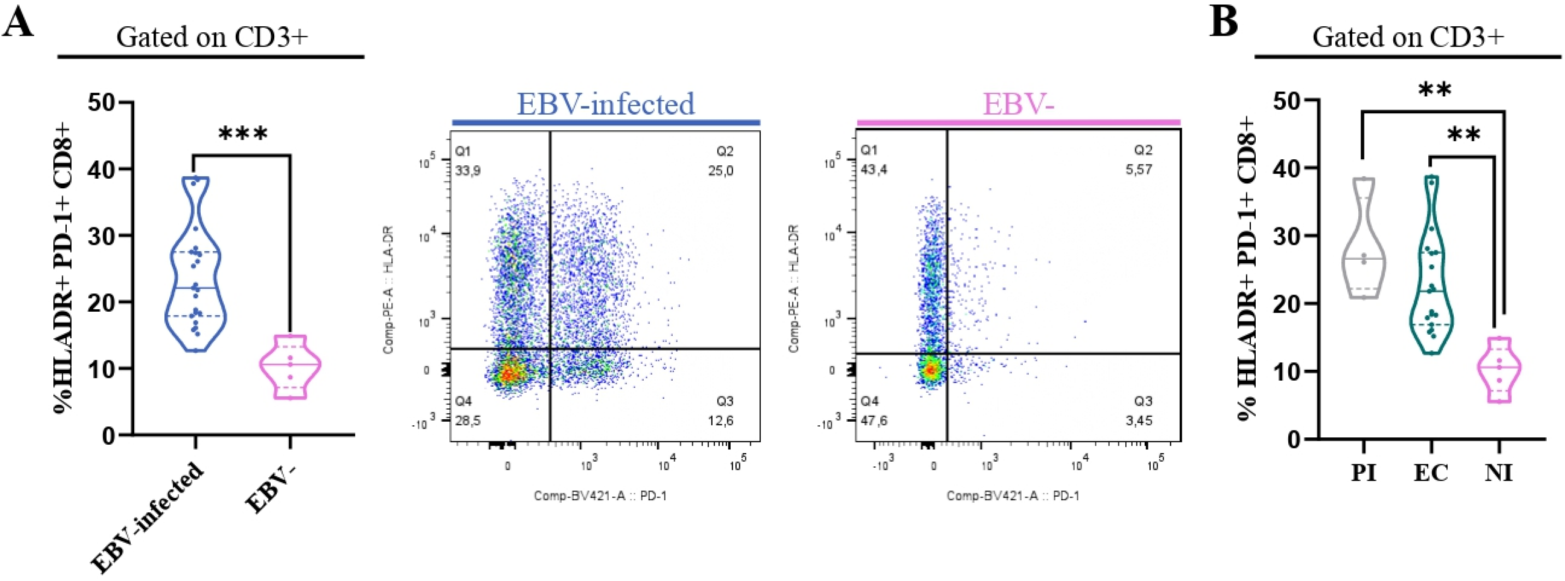
HLA-DR+PD-1+ CD8+ T cells. Violin plot on the left of PD-1+ HLADR+CD8+ T cells in EBV-infected (EI) and non-infected (NI) patients and representative dot plots on the right **(A)** and violin plots of PD-1+HLADR+ CD8+ T cells for PI patients, EC and NI **(B)**. **p<0.01, ***p<0.001.

To determine whether activated CD8+ T cells also co-express PD-1, their frequency across different EBV infection statuses was analyzed, aiming to assess if these cells exhibit features of exhaustion. First, we compared the frequency of HLA-DR+ PD-1+ CD8+ between EBV-infected patients and EBV-. Significantly, the percentage of HLA-DR+ PD-1+ CD8+ T cells was higher in EBV-infected patients compared with NI ones (p=0.0007, *t*-test) (**Figure 5A**). In addition, we evaluated how these cells behaved over the different EBV statuses and we observed a significantly higher frequency of HLA-DR+ T PD-1+ CD8+ T cells in PI patients and EBV carriers compared with NI (p =0.002 and p=0.004, respectively; ANOVA followed by Tukey’s test) (**Figure 5B**).

### 3.5 Memory T cells in persistent EBV-infection

**Figure 6:**
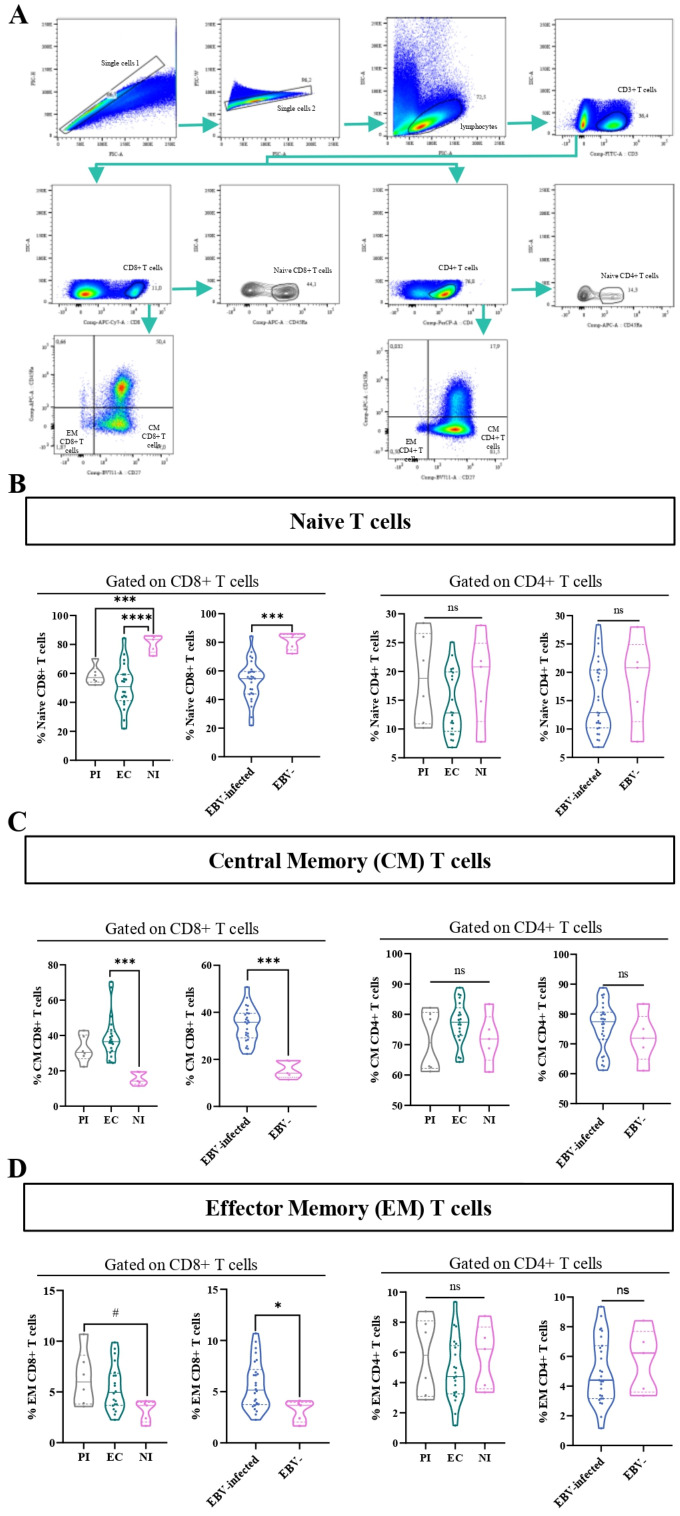
Memory and naive T cells. Representative gating strategy for analyzing naive, central memory (CM), and effector memory (EM) CD4+ and CD8+ T cells **(A)**. Violin plot of naive CD8+ and CD4+ T cells for PI patients, EC, and NI, and EBV-infected (EI) and non-infected (NI) patients **(B)**. Violin plot of CM CD8+ and CD4+ T cells for PI patients, EC, and NI, and EBV-infected (EI) and non-infected (NI) patients **(C)**. Violin plot of EM CD8+ and CD4+ T cells for PI patients, EC, and NI, and EBV-infected (EI) and non-infected (NI) patients **(D)**.# trend *p<0.05, ***p<0.001 **** p<0.0001, ns: not significant.

CD45RA along with the costimulatory molecule CD27 was used to classify T-cell subsets (CD4+ and CD8+) into various stages of differentiation, such as naive (CD45RA+), central memory (CM) (CD27+CD45RA−), and effector memory (EM) (CD27−CD45RA−), as previously described (17). The gating strategy to analyze the percentage of these cells is shown in **Figure 6A**. The frequency of naive CD8+ T cells was lower in PI and EC compared with NI (p=0.0006 and p<0.0001, respectively, ANOVA, followed by Games–Howell’s multiple comparisons test), and, as expected, this observation was confirmed comparing EBV-infected with NI patients (p=0.0002, t-test). On the other hand, no statistical differences in the frequencies of naive CD4+T cell was observed among EC, PI, and NI, as well as between EBV-infected and NI children (p>0.05, KW and MW tests, respectively) (**Figure 6B**). In contrast, the frequency of CM CD8+ T cells was higher in EC and PI concerning NI (p=0.0008 and p=0.0773, respectively, KW followed by Dunn’s test). In line with this, when EBV-infected and NI patients were compared, the frequency of CM CD8+ T cells was higher in EBV-infected children versus NI ones (p<0.0001, t-test). By contrast, no statistical differences in the frequency of CM CD4+T cells was observed among EBV infection status, and comparing EBV-infected vs. NI groups (p>0.05, KW and t-tests, respectively) (**Figure 6C**). In addition, a trend to a higher frequency of EM CD8+ T cell was found in PI concerning NI (p=0.0611, ANOVA, followed by Tukey test) and turned into a significant increase in EBV-infected compared with NI (p=0.0281, t-test). By comparison, no statistical differences in the frequency of EM CD4+T cells were observed among EBV status infection and between EBV-infected vs. NI children (KW and t-tests, respectively) (**Figure 6D**).

### 3.6 Implications of EBNA2 and LMP1 expression in CD4+ and CD8+ T subpopulations

CD4+ follicular helper T cells (Tfh) are essential in the formation of the GC, which is the histological site where most B-cell lymphomas, including those associated with EBV, are generated (18). Additionally, the germinal center model (GCM) explains that EBV needs to transit the GC to access the resting memory compartment, and LMP1 as well as EBNA2 expression at this histological site was described (19). As a result, immune deregulation in that compartment could lead to the development of the different diseases EBV-associated (4). Within those cells, CD4+ follicular regulatory T cells (Tfr) are a specific subset that control GC cell number and Tfh function (20). Thus, one of our aims was to investigate the behavior of these populations across different infection statuses and latency profiles, as well as to explore potential correlations between these populations and the expression of main viral latency proteins, EBNA2 and LMP1.

**Figure 7:**
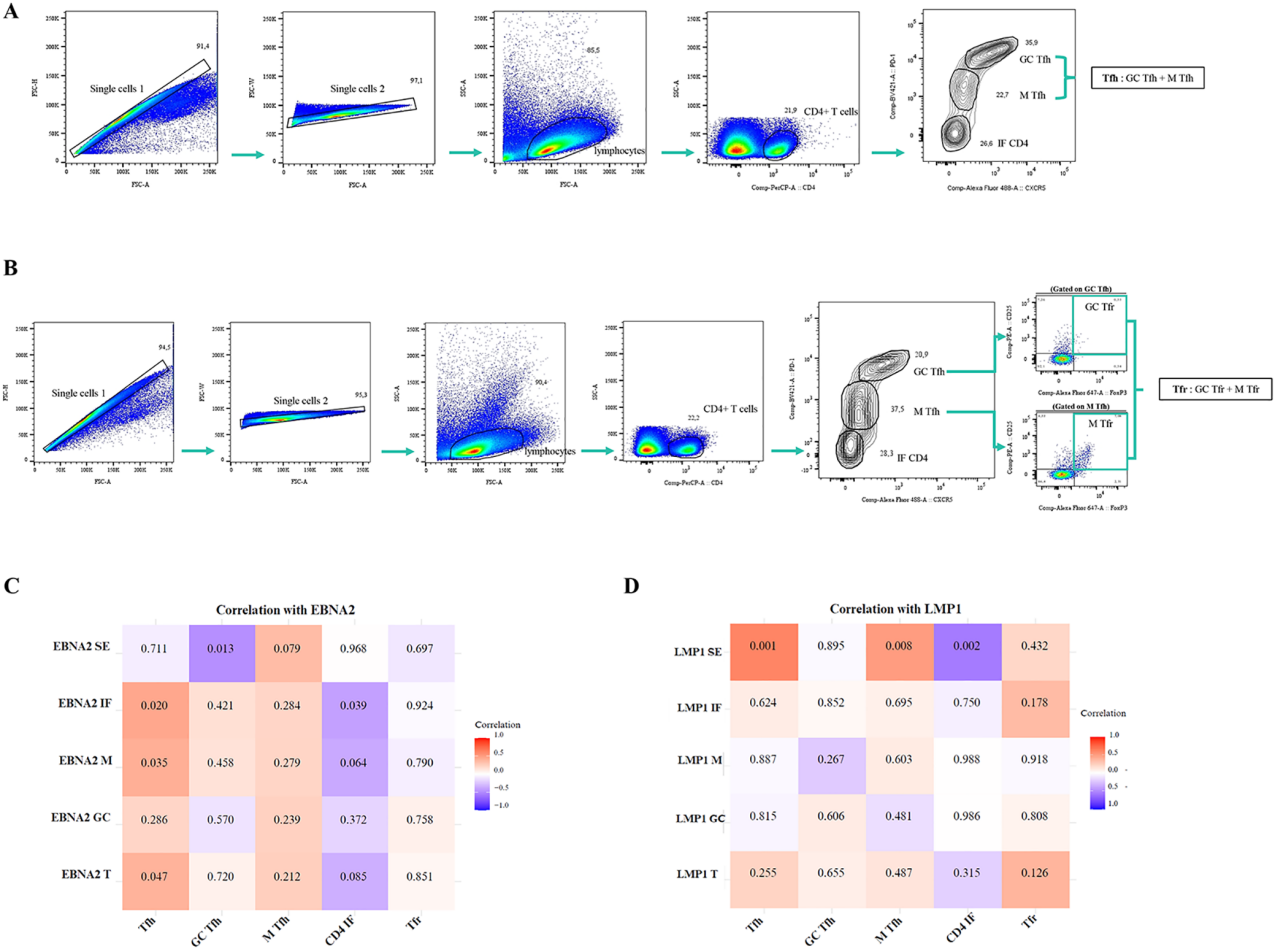
Correlation of EBNA2 and LMP1 in distinct histological regions of the tonsil with Tfh, GC-Tfh, M-Tfh, CD4 IF, and Tfr cells. Representative gating strategy for analyzing Tfh (GC-Tfh+M-Tfh), GC-Tfh, M-Tfh, and CD4 IF cells **(A)**. Representative gating strategy for analyzing Tfr (GC-Tfr + M-Tfr) cells **(B)**. Matrix correlation of EBNA2 SE, IF, M, GC, and T with Tfh, GC Tfh, M Tfh, CD4 IF, and Tfr cells **(C)**. Matrix correlation of LMP1 SE, IF, M, GC, and T with Tfh, GC Tfh, M Tfh, CD4 IF, and Tfr cells **(D)**. The heatmap represents the correlation values, and the numbers inside each grid indicate the p-values. SE, subepithelial; IF, interfollicular; M, mantle; GC, germinal center.

The gating strategy to analyze Tfh (GC+ M), GC-Tfh, M-Tfh, CD4 IF, and Tfr is shown in **Figure 7A, B**. These populations were phenotypically classified using the surface markers CXCR5 and PD-1, as well as the intracellular marker FOXP3 and the surface marker CD25 to define Tfr. Therefore, we categorize the population of CD4+ as follows: Tfh (CXCR5+PD-1+), GC-Tfh (CXCR5^hi^PD-1^hi^), M-Tfh (CXCR5^int^PD-1^int^), CD4 IF (CXCR5^low^PD-1^low^), and Tfr (CXCR5+PD-1+FOXP3+CD25+) defined as the sum of GC-Tfr (CXCR5^hi^ PD-1^hi^FOXP3+CD25+) and M-Tfr (CXCR5^int^PD-1^int^FOXP3+CD25+).

When patients by infection status and by different latency profiles were compared, there were no differences in the percentages of Tfh, GC-Tfh, M-Tfh, CD4 IF, and Tfr cell populations (p>0.05, MW test). A positive correlation was observed between EBNA2 T cells with a frequency of Tfh (p=0.047, R=0.3928, Spearman correlation). Moreover, this positive correlation was also maintained in EBNA2 located in the IF and M regions (p=0.020, R=0.453 and p=0.035, R=0.415; respectively, Spearman correlation). In contrast, a negative correlation was detected between EBNA located in the IF region and the percentage of CD4 IF (p=0.039, R=−0.407, Spearman correlation), as shown in the heatmap (**Figure 7C**).

On the other hand, there was a positive correlation of Tfh and M-Tfh with LMP1 SE (p=0.001, R=0.620 and p=0.008, R=0.507; respectively, Spearman correlation) and a negative correlation of CD4 IF with LMP1 SE (p=0.002, R=-0.573, Spearman Correlation), as shown in the heatmap (**Figure 7D**).

**Figure 8:**
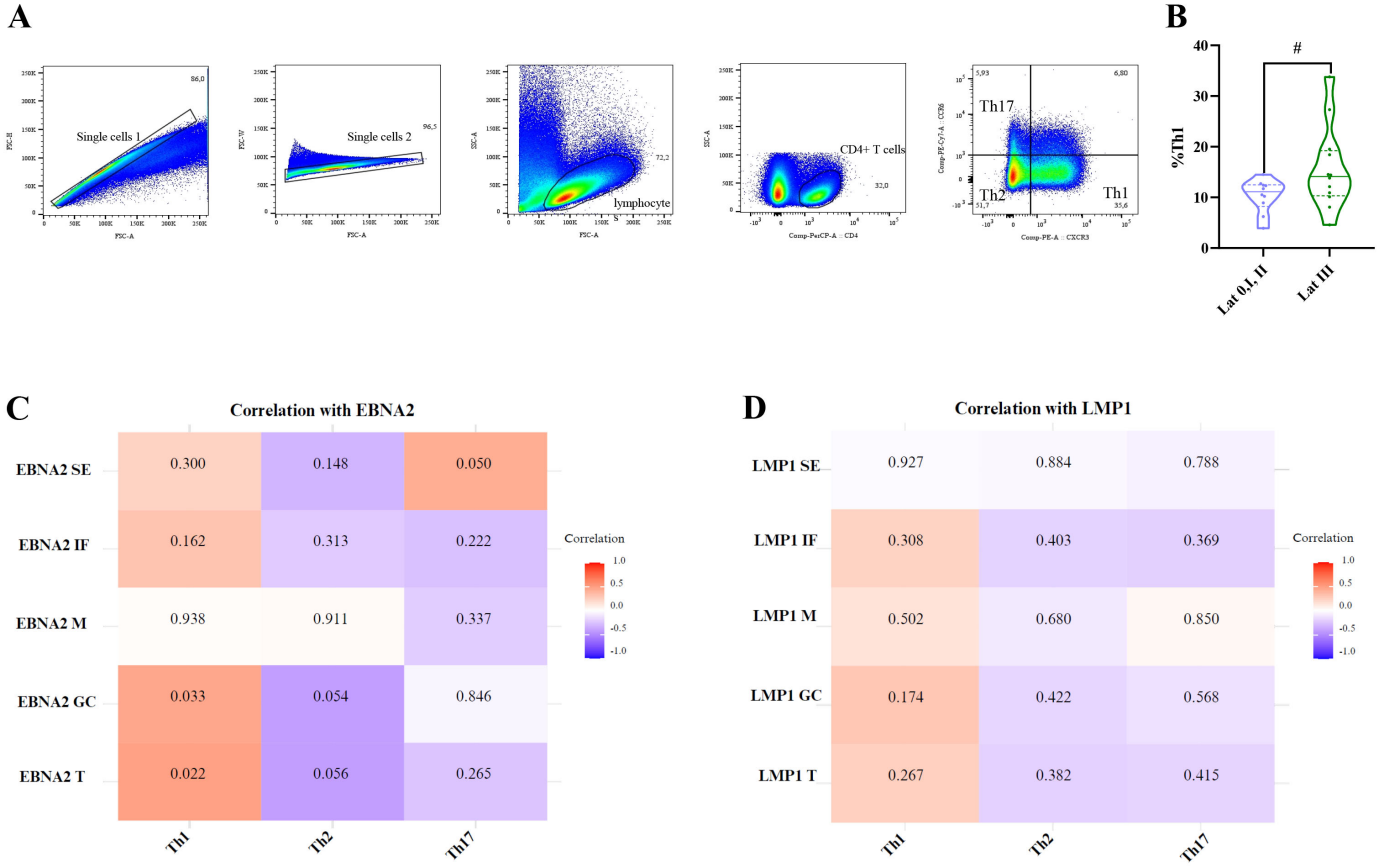
Correlation of EBNA2 and LMP1 in distinct histological regions of the tonsil with Th1, Th2, and Th17 cells. Representative gating strategy for analyzing Th1, Th2, and Th17 cells **(A)**. Violin plot of Th1 for latency 0+I+II patients and latency III patients **(B)**. Matrix correlation of EBNA2 SE, IF, M, GC, and T with Th1, Th2, and Th17 cells **(C)**. Matrix correlation of LMP1 SE, IF, M, GC, and T with Th1, Th2, and Th17 cells **(D)**. The heatmap represents the correlation values, and the numbers inside each grid indicate the p-values. SE, subepithelial; IF, interfollicular; M, mantle; GC, germinal center # trend.

CXCR3 and CCR6 were used to study the other CD4+ T-helper cell (Th) populations, in relation to the different latency profiles and infection statuses. These populations were characterized as follows: Th1 (CXCR3+CCR6−), Th2 (CXCR3-CCR6−), and Th17 (CXCR3-CCR6+), as previously described (21). The gating strategy is shown in **Figure 8A**.

Consistent with the observations made for follicular CD4+T cell populations, when patients were compared by infection status, no significant differences were observed in the percentages of Th1, Th2, and Th17 populations (p>0.05, MW test). However, patients with a latency III profile (latency 0, I, and II combined vs. latency III) demonstrated a trend toward a higher percentage of Th1 cells (p=0.0583, t-test) (**Figure 8B**). In line with this, upon the correlations between these populations and the expression of LMP1 and EBNA2 latent proteins, only Th1 correlated with total EBNA2 cell count (EBNA2 T) (p=0.0218, R=0,4861, Spearman Correlation), and this correlation was maintained for EBNA2 in the GC (p=0.033, R=0.455, Spearman correlation) (**Figures 8C, D**).

**Figure 9:**
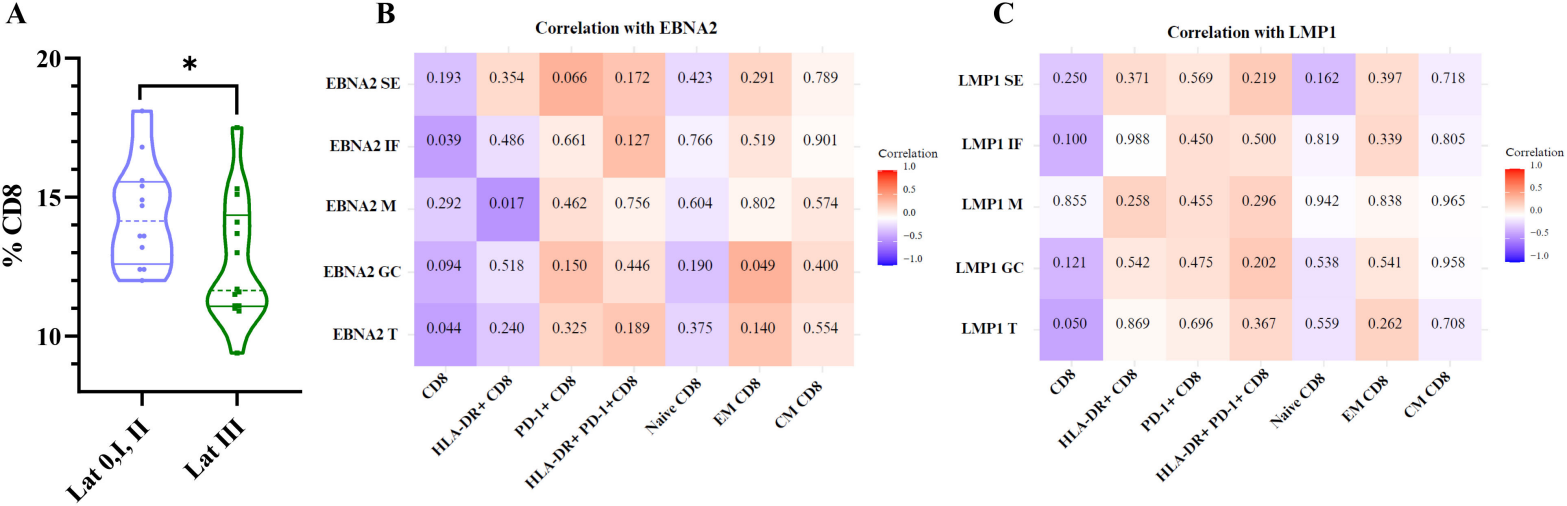
Correlation of EBNA2 and LMP1 with CD8, HLA-DR+ CD8, PD-1+CD8, HLA-DR+ PD-1+ CD8, naive CD8, EM CD8, and CM CD8+ T cells. Violin plot of % CD8 for latency 0+I+II patients and latency III patients **(A)**. Matrix correlation of EBNA2 SE, IF, M, GC, and T with CD8, HLA-DR+ CD8, PD-1+CD8, HLA-DR+ PD-1+ CD8, naive CD8, EM CD8, and CM CD8 T cells **(B)**. Matrix correlation of LMP1 SE, IF, M, GC, and T with CD8, HLA-DR+ CD8, PD-1+CD8, HLA-DR+ PD-1+ CD8, naive CD8, EM CD8, and CM CD8+ T cells **(C)**. The heatmap represents the correlation values, and the numbers inside each grid indicate the p-values. SE, subepithelial; IF, interfollicular; M, mantle; GC, germinal center *p<0.05.

Finally, since several subpopulations of CD8+ T cells varied according to the different EBV infection statuses, we decided to evaluate whether there were differences in the percentages of these populations across the different latency profiles, as well as their correlation with LMP1 and EBNA2. Patients with a latency III profile exhibited a lower percentage of total CD8+ T cells (p=0.042, t-test) compared with latency 0/I and II patterns (**Figure 9A**). However, no significant differences were observed in the percentages of activated CD8+, PD-1+CD8+, naive CD8+, CM CD8+, and EM CD8+ T cells across the different latency profiles (p>0.05, t-test).

Consistently, when analyzing the correlations between these populations and the expression of the latent proteins LMP1 and EBNA2, the total CD8+ T cells showed a negative significant correlation with total EBNA2 cell count (EBNA2 T) (p=0.044, R=−0.405 Spearman correlation) and HLA-DR+ CD8+ T cells with EBNA2 M and positive correlation between EM CD8+ T cells with EBNA2 GC as shown on the heatmap (**Figures 9B, C**).

## 4 Discussion

Many studies focus on investigating the behavior of CD8+ and CD4+ T cells in peripheral blood in the context of a symptomatic primary infection, such as IM (22), but local immune response at the site of primary EBV infection, the tonsils, is still not fully understood, particularly in children with asymptomatic infection. In a preliminary analysis to understand the balance between EBV and the immune system to avoid malignant transformation, our group demonstrated an increase of CD8+ and CD4+ T cells at the subepithelial and germinal center regions, respectively (23), along with an increase in cytotoxic CD4+ T cells in children with primary infection (15). To further explore these findings across different EBV infection statuses and latency profiles, local CD4+ and CD8+ T cells were analyzed in a pediatric cohort.

A peripheral massive expansion of activated CD8+ T cells, particularly in response to lytic antigens, is characteristic of IM and is also responsible for the disease symptoms (11). In five cases with asymptomatic primary infection in young adults, peripheral CD8+ T cell-mediated responses, even where they resemble IM in timing and quality, were never as exaggerated (24). Regarding local immune response, a predominance of CD8+ over CD4+ T cells was previously reported in the tonsils of adults with IM in a developed population (25). In line with this, in this study, we described not only a higher percentage of CD8+ T cells over CD4+ T cells in EBV-infected cases but also a particular increase of CD8+ T cells in children with asymptomatic primary infection. Moreover, primary infected children are characterized by exhibiting a locally higher percentage of activated CD8+ T cells in the tonsils, similar to what was previously described in the peripheral blood of pediatric patients with IM. However, in contrast to what has been described in children with IM, the presence of activated marker HLA-DR in CD8+ T cells is not associated with the severity of symptoms, as the infection in the analyzed cohort is asymptomatic (26). The lack of symptoms in our cohort could be partially explained by the increase of PD-1+ CD8+ T cells in EBV-infected cases, which may counterbalance the effect of cytotoxic cells. PD-1 expression was markedly upregulated on tumor infiltrating CD8+ T cells and correlated with reduced cytokines in several types of cancers, such as Hodgkin lymphoma (27). In the context of lymphocytic choriomeningitis virus infection, even though virus-specific CD8+ T cells initially develop the ability to perform effector functions, these functions are lost in a hierarchical manner during chronic infection, since some functions are exhausted early, whereas others (e.g., IFN-γ) persist longer (28). In our series, upregulation of PD-1 expression, in CD8+ T cells and activated CD8+ T cells, was demonstrated not only during primary infection but also in EBV carriers. In line with this, an increase of PD-1 expression was demonstrated in TEMs from six adult EBV-infected donors undergoing tonsillectomy and suggested that, upon EBV primary infection, T cells express inhibitory molecules including PD-1 and LAG-3, which could be used by EBV-infected B cells to inhibit their function (10). Therefore, in our cohort, PD-1 expression maintained during primary as well as persistent infection may suggest that CD8+ T cells gradually upregulate PD-1 during the process of EBV persistence, exhibiting features suggestive of exhaustion or functional modulation. However, functional studies are required to confirm the presence of exhaustion in CD8+ T cells.

The effect of EBV infection on the T-cell compartment in children was characterized by an expansion of CM CD4+ T cells and an increase in EM CD8+ T cells, whereas naive CD8+ T cells and CM CD8+ T cells remained unchanged in peripheral blood (29). In children, a decline in memory CD8+ T cells has been reported during the early acute stage of infectious mononucleosis (30). In our tonsillar cohort, EBV-infected children displayed an increase in CM and EM CD8+ T cells, along with a decrease in the naive population, whereas the corresponding CD4+ T-cell populations remained stable, all compared with non-infected children. The increase in CM CD8+ T cells was observed in both PI children and EBV carriers, whereas EM CD8+ T cells were particularly elevated in PI individuals. CM T cells are a small population of memory T cells that circulate between the secondary lymphoid organs and the blood. They are long-lived and can be activated rapidly upon reencountering their cognate antigen in secondary lymphoid organs. EM T cells have been proposed to migrate through the blood, as well as lymphoid and non-lymphoid tissues, to kill pathogens via a variety of effector mechanisms, and gradually disappear once the pathogens have been eliminated (31). The presence of EM CD8+ T cells, particularly in PI within our cohort, may reflect their recruitment for the efficient elimination of EBV in recently infected children. Meanwhile, the presence of CM CD8+ T cells in both PI and EC may indicate ongoing surveillance for EBV reactivation, allowing for rapid activation upon the appearance of viral antigens.

GCs are the histological structures dedicated to the generation and the selection of B cells that produce high-affinity antibodies. The majority of B-cell non-Hodgkin lymphomas, including Burkitt lymphoma, follicular lymphoma, and diffuse large B-cell lymphoma, as well as Hodgkin lymphomas, are derived from GC B cells, as shown by the presence of somatically mutated immunoglobulin genes in their genomes (32, 33). EBV takes advantage of the GC reaction during infection to persist for life in memory B cells ultimately. Therefore, the GC are key structures to study EBV-associated B-cell lymphomas. Moreover, it was demonstrated that the T-cell response plays a key role in EBV-mediated lymphomagenesis in GC. In fact, in a mouse model, the depletion of T cells in GCs expressing both LMP1 and LMP2A viral proteins led to fatal lymphoma in all mice (34). Furthermore, Tfh cells are associated with the development of EBV-associated lymphoma (18), and depletion of Tfh as well as Tfr cells delayed the onset of lethal EBV-associated lymphoproliferative disease in mice and improved survival (35). Moreover, activation of the IL-6/STAT3 signaling pathway was demonstrated, leading to the secretion of IL-21, a signature cytokine of Tfh cells, which promotes B-cell activity (35). These findings support the notion that Tfh cells may contribute to the enhanced proliferative capacity of EBV-infected B cells. In addition, immune pressure has been proposed as a driving force behind the transition to the EBV latency II program at the GC, in which EBNA1, LMP1, and LMP2A are expressed (36). Tfh cells promote rapid GC formation and antibody production in acute infections, whereas their dysfunction in chronic infections can impair immune responses and contribute to viral persistence (37). In the context of EBV infection, the role of CD4+ T cells in the regulation of EBNA2 was previously demonstrated *in vitro* (38). Furthermore, the effect of cytokines secreted by GC-resident Tfh cells in repressing latency III EBNAs and supporting LMP1 expression, key events in the transition from latency III to latency II, has been demonstrated (39). Circulating Tfh and Tfr are increased in peripheral blood in adults with IM, suggesting an imbalance of circulating Tfr and Tfh cells as responsible for the immunopathogenesis of IM (40). In our cohort, the absence of differences in tonsillar Tfh and Tfr cells might be related to the absence of symptoms. In addition, a positive correlation between EBNA2 expression in the IF and M regions and Tfh cells was observed, but not in the GC. Even though causality cannot be inferred from correlation analysis alone, this finding might partially explain that EBNA2 expression in the GC could result from inefficient recruitment of Tfh cells, which would otherwise downregulate EBNA2 within the GC. This finding is reinforced by the previous observation of latency III antigens at the GC in children infected with EBV (19). The downregulation of latency III profile antigens might be mediated only by CD4+ cytotoxic T cells, as previously suggested by our group (15). Even though LMP1 expression in B cells has been shown to induce potent cytotoxic CD4+ T-cell responses (41), in our series, LMP1 expression may not play an indirect role in modulating either CD4+ or CD8+ T cells. In fact, only LMP1+ cells in the subepithelial region may influence CD4+ T cells, consistent with our previous study, which attributed a role to LMP1+ cells in the increase of CD8+ T cells and granzyme B+ cells in the tonsillar subepithelial region in children (23).

In Argentina, the increased incidence of EBV-associated lymphomas in children younger than 10 years was demonstrated (12). This study provides insights into the behavior of different subpopulations of total CD4+ and CD8+ cells in the tonsils, the main site of viral entry and reactivation, of children infected with EBV. Although this hypothesis is speculative, the upregulation of PD-1 expression, only in CD8+ T cells, during persistent infection, may suggest features indicative of exhaustion or functional modulation during the process of EBV persistence. In addition, the expression of certain oncogenic proteins such as EBNA2, which might be relatively resistant to downregulation by Tfh cells, may contribute to malignant transformation, ultimately triggering an EBV-associated lymphoma derived from the GC in certain individuals. To finally confirm a causal relationship, further studies in other populations, as well as functional studies, are required to support these findings.

## Data Availability

The raw data supporting the conclusions of this article will be made available by the authors, without undue reservation.
