# Supplementary material for: Co-expression of PD1+ and HLA-DR+ in CD8+ T cells is increased in tonsils of children with EBV primary and persistent infection
Source: Front Immunol. 2025 Sep 2;16:1653165. doi: 10.3389/fimmu.2025.1653165 (PMC12436462; doi:10.3389/fimmu.2025.1653165)
Supplement: Supplementary file 1 [file Table1.docx]

Supplementary Material

# Supplementary Tables

**Supplementary Table 1.**


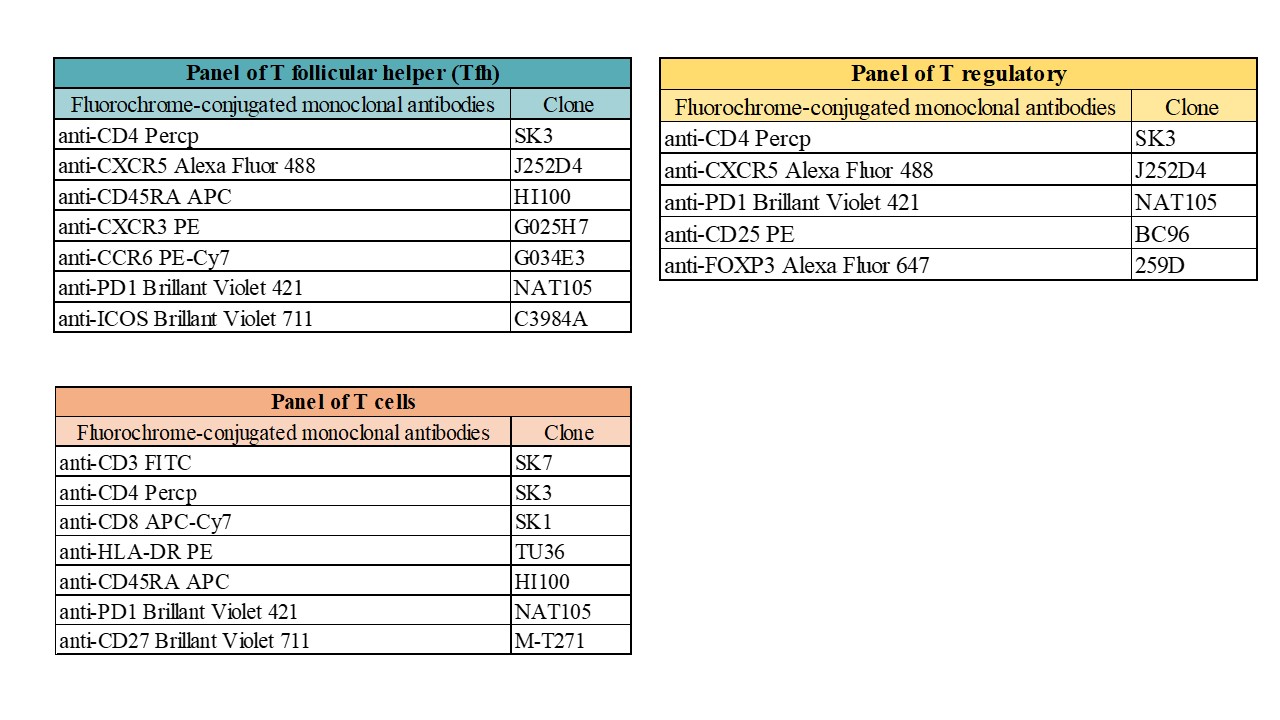


*all antibodies are Biolegend

**Supplementary Table 2.**

**Supplementary figures
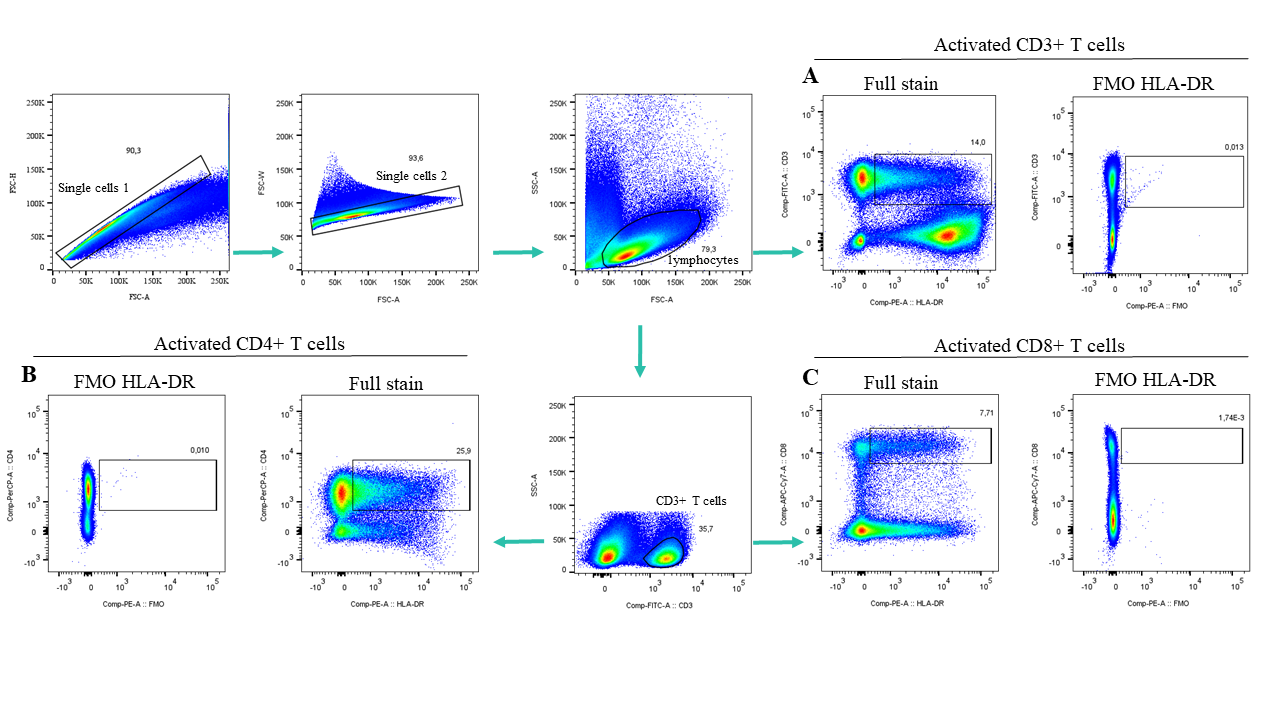
**

**Supplementary Figure 1.** Gating of Activated CD3+ **(A)**, CD4+ **(B)**, and CD8+ **(C)** T cells. For panels **A** and **C**, the full stain is shown on the left and FMO HLA-DR control on the right. For panel **B** (activated CD4+ T cells), the FMO HLA-DR control is shown on the left and the full stain on the right. Gates for HLA-DR+ cells were defined using the FMO and applied to the full stain samples.
